# Hardening of Bimetallic Wires from Secondary Materials Used in the Construction of Power Lines

**DOI:** 10.3390/ma15113975

**Published:** 2022-06-02

**Authors:** Irina Volokitina, Natalia Vasilyeva, Roman Fediuk, Alexandr Kolesnikov

**Affiliations:** 1 Department of Metallurgy and Mining, Rudny Industrial Institute, 111500 Rudny, Kazakhstan; irinka.vav@mail.ru; 2 Mineral Raw Material Processing Faculty, Saint Petersburg Mining University, 199106 St. Petersburg, Russia; 3 Polytechnic Institute, Far Eastern Federal University, 690922 Vladivostok, Russia; 4 Peter the Great St. Petersburg Polytechnic University, 195251 St. Petersburg, Russia; 5 Department of “Life Safety and Environmental Protection” M. Auezov, South Kazakhstan University, 160012 Shymkent, Kazakhstan

**Keywords:** severe plastic deformation, bimetallic wire, steel-copper wire, microstructure, mechanical properties

## Abstract

Copper-sheathed steel wires combine the conductivity of copper and the traction resistance of steel, which makes a bimetallic wire an ideal material for the construction of power lines. Currently, there is a small number of studies devoted to the change in the microstructure of steel-copper wire during its strain. Since steel and copper have different mechanical properties, these metals at the interface can be deformed in different ways. Therefore, the present research is devoted to the study of ECAP-drawing process impacts on the properties of bimetallic steel-copper wire. During the conducted studies, the possibility and efficiency of using the combined strain technology for the formation of ultrafine grained structure and increased strength properties of steel-copper wire have been proved.

## 1. Introduction

Power lines and cables reinforced in the upper part of transmission line supports used for the protection of wires from atmospheric over-voltages and direct lightning strikes [1,2,3,4,5] work in harsh conditions because they are located outdoors and exposed to various atmospheric phenomena (wind, rain, ice, and temperature changes) and chemical impurities in the surrounding in the air [6,7,8,9,10,11]. In this regard, the wires must have sufficient mechanical strength and withstand the effects of atmospheric phenomena and chemical impurities along with possessing good electrical conductivity. Therefore, in recent years, during the construction of power lines, much focus has been directed toward bimetallic materials formed by a combination of metals dissimilar in their layered structure [12,13,14]. The main quality indicators of bimetallic wire and rods are the combination of their strength and plastic properties. At the same time, the main properties of bimetallic material are structurally sensitive, i.e., they can be controlled by purposeful changes in the structure when it is processed by pressure [15,16,17,18]. Bimetal wire with a steel core and different sheaths comprise copper, aluminum, and brass sheaths that are widely used in the industry. Such wires have increased corrosion resistance and most often possess good electrical conductivity and high strength. Although, currently, the price level of copper is constantly changing, it remains the dominant metal in the cable industry. A copper shell provides low electrical resistance while the steel core provides high structural strength, which can be modified by carbon contents and heat treatment [19,20]. 

Despite the fact that the technologies that are used for solid-phase joining of several dissimilar metals develop every year [21,22,23,24], it is almost impossible to increase the strength properties of steel metal due to a number of technological features. These features include the formation of chemical compounds on the surface of copper sheath during heating and the embrittlement of bimetallic wires when heated and passed through an alcohol solution for clarification. Most importantly, using the more expensive steels reduces the economic efficiency of the entire production process. 

One of the most promising areas in terms of increasing the plasticity and strength properties of steel-copper wire is the obtainment of ultrafine-grained wire in such wires by using pressure metal treatment (PMT) methods [25,26,27,28,29,30]. Since heating a steel–copper wire composed of two metals with different physical and mechanical properties can lead to diffusion processes, this can result in the formation of brittle intermetallic inclusions at the boundary of the steel–copper bond, which hinders the subsequent use of such a wire. In this regard, severe plastic deformation (SPD) methods that implement a simple shear scheme under multicyclic processing conditions are being actively developed. Obtaining ultrafine grained structure in such metals allows an increase in strength and plastic properties of the respective metallic materials [31,32,33,34,35]. Based on the above, we can conclude that the search for new and productive schemes for the formation of ultrafine grained structure in metals and alloys on the basis of already available traditional PMT methods is the most important step for the further development of all processes related to severe plastic deformation.

Equal-channel angular pressing (ECAP), which is probably the most widely used SPD technology, is considered as a promising method for achieving the desired high degree of strain [36,37,38,39]. Recently, based on this method, a large number of its modifications have appeared [40,41], some of which render it possible to obtain long-lasting products [42,43]. Raab et al. [44] combined ECAP with Conform to continuously produce ultrafine-grained materials of aluminum. The method provides high material utilization while obtaining long-length semi-finished products and a high level of physical and mechanical properties. However, it does not exclude the need for multicyclic processing, which causes an increased complexity of the process and increases the cost of energy. To overcome such drawbacks of previous research, Hwang et al. [45] recently proposed a shear drawing (SD) process that has a cone-shaped channel and corner radius at the intersection. The SD process ensured stable flow and imposed relatively high plastic deformation on the material as a continuous process. However, it was reported in their work that a smaller intersecting angle may decrease flow stability and cannot easily guarantee the dimensional accuracy of the diameter due to tension, which causes undesirable reductions in the area of the deformed sample.

The extruding process was proposed in 1976 by Avitzur [46]. This method combines rolling and pressing actions at one strain center. The main difference between this method and others is lower power loss due to reactive friction and a more complete filling of the gauge cavity with the metal materials of the workpiece. The main imperfection of this process is the limited length of the resulting workpiece and the small draft. Since this method does not create the necessary pressure required for extrusion and does not provide a steady flow of metal, it has not found further industrial applications. 

In the USA, WesternElectricCo specialists proposed a method called Lainex. In this method, continuous pressing takes place due to active friction forces. The active friction forces occur between the upper and lower planes of the workpiece, which has a rectangular cross-section and the flat surfaces of the chain links. The pressing force, in this case, depends on the difference between the frictional forces on the unlubricated and lubricated planes of the workpiece. This method is used for wire processing and aluminum tire production at Wenskak plants. Compared to the Conform method, the maximum value of the elongation coefficient in this method is by an order lower, which is the main disadvantage of this process.

The extraction process has been modernized and developed as a combined rolling and pressing (CRP) method. This method is formed on the strain of a long workpiece that has a rectangular cross-section in a closed gauge of a two-roll type, which is blocked at the output by a die [47].

For obtaining lengthy sizes, combined processes were developed in which equal channel angular pressing is carried out jointly with drawing processes. Many of these methods are based on bending, for example, multiple angular storage drawing described in the work of Muszka et al. [48] and a method for producing long-length workpieces of circular cross section proposed by Chukin et al. [49]. However, from our point of view, during the production of long products, it is inappropriate to use methods based on bending due to imperfection of rigging and the difficulty of integrating it into existing equipment. The most promising methods are based on torsion. The method proposed by Chukin et al. [50] constitutes a method continuously strain nanostructuring wire using additional shear strain by alternating bending and torsion. Equipment for the implementation of this method is well integrated into the line of drawing machines. However, this method requires additional equipment, and the degree of accumulated shear strain is limited by the high probability of fractures in the metal under torsion. In the method proposed by Raab et al. [51], wire drawing is performed with a shift of two successively arranged drawing dies, the internal channel of which is made in the form of a displaced cone wherein the drawing die at the output rotates about its axis. However, the application of this method in industrial applications is limited to the complexity of making drawing dies. The method of equal-channel angular free drawing should be noted, which leads to the formation of ultrafine-grained (UFG) structure in long workpieces in circular cross-section. Chukin et al. [52] showed that it is necessary to carry out the method from 4 to 10 processing cycles in order to obtain the UFG structure, which is the main disadvantage of this process. Moreover, when ECAP drawing occurs in the deformed workpieces, a heterogeneous ultrafine grain structure is observed even after eight passes over the entire cross-section.

However, despite numerous developments, the problem of creating a new strain scheme that is as close as possible to industrial conditions and is of scientific and practical interest from the point of view of mass production remains unresolved. One of these schemes can be a new continuous scheme of equal-channel angular pressing and drawing (ECAP-drawing) method [53,54,55,56]. Its key feature is that, unlike other combined methods, there is no rolling stage. The continuity of strain is ensured by the drawing process, which occurs immediately after the ECAP process. Due to such a unique strain scheme, a sufficiently high level of tensile stresses develops in the cross section of the workpiece.

Therefore, the purpose of this study is to obtain bimetallic steel–copper wires used in the construction of power transmission lines with ultrafine grain structure and increased strength characteristics. 

## 2. Materials and Methods

A bimetallic wire consisting of steel core-steel 1566 (Fe; 0.65-C; 0.21-Si; 1.1-Mn; 0.2-Ni; 0.2-Cr, weight%) and an outer layer of copper M1 (99.9 Cu) with a diameter of 10 mm was used as the initial workpiece; the diameter of the steel core was 8 mm.

The simplest technical solution for the creation of an experimental installation for the implementation of “ECAP-drawing” combined process was the creation of an installation on the basis of an industrial drum drawing mill V-1/550M, which is additionally equipped with an equal-channel stepped matrix, which makes it possible to implement severe plastic deformation during strain processes. An equal-channel stepped die is located in the lubrication container in front of the wire holder. In order to prevent wire breakage, a sharpening machine was used as a wire feeder in the combined process. This machine is an auxiliary equipment of drawing mill. The rotation of rollers was set so that active friction forces fed the wire into the equal-channel stepped matrix and prevented the emergence of excessive wire tension forces. Soap shavings were used as a lubricant. After the pre-sharpened end of wire has been set in an equal-channel stepped matrix and in drawing frame, i.e., the end of wire left drawing frame, it was fixed by drawing frame pincers. After safety door closing, the drawing mill was switched on and the wire, which has been deformed in combined “ECAP-drawing” processes, is spooled onto the drawing drum. After each deformation run, die and drawing die were changed to a smaller diameter. The strain was performed at room temperature; the number of strain cycles was 3. The technology and conditions of strain are described in detail in [47]. In the first stage, the workpiece passed through die channels with a diameter of 10 mm, and in the drawing stage, the diameter was reduced by 5% (9.7% compression) to a diameter of 9.5 mm. In the second stage, the workpiece passed through the die channels with a diameter of 9.5 mm, and in the drawing stage, the diameter was reduced by 5.26% (10.2% compression), to a diameter of 9 mm. In the third stage, the workpiece passed through the die channels with a diameter of 9 mm, and in the drawing stage, the diameter was reduced by 11% (compression 10.8%) to a diameter of 8.5 mm.

A fine structure was studied on a transmission electron microscope (TEM) JEM2100 in the magnification range from 1000 to 50,000 times. The subjects for TEM were prepared by polishing with a Tenupol-3 device at a temperature of −28 °C and a voltage of 20 V in a 20% solution of nitric acid in methyl alcohol. For the purpose of a more objective interpretation of the grain structure compared to TEM, EBSD analysis was performed using a Philips XL-30 REM with a field cathode. The accelerating voltage is 20 kV. Tex SEM Lab software was used to process the results. The misorientation was calculated between neighboring (adjacent) scan points. The dimensions of the scan step were previously determined from the measurement areas and the expected grain sizes or subgrains. Scanning was carried out on sections of 50 μm × 50 μm in 0.2 μm increments. Various misorientations between the grains were established using the minimum resolution of misorientation 2°. Taking into account the experimantal accuracy of the EBSD method, all small-angle boundaries with a misorientation of less than 2° have been excluded from consideration. All scanned reference points with a confidence index of ~0.1 were excluded from the sets, and this was performed to improve the overall accuracy of images. The colors of grains on the map correspond to the orientations indicated in the stereographic triangle. Thus, different colors in the neighboring grains correspond to the misorientation between these two grains more than 2°. The grain boundaries are indicated either by white lines corresponding to small-angle misorientations 2–15° or by black lines corresponding to high-angle misorientations >15°. The fraction of indexed diffraction patterns was 98% of the total number of measured points. On all EBSD cards, all points that were unindexed were removed during the standard replacement (cleaning) procedure. The surface of workpieces was prepared by jet polishing on the Tenupol-3 device.

Tensile mechanical tests of bimetallic wire were carried out using standard methods on an Instron 5982 electromechanical measuring machine. The wire tensile speed was 0.5 mm/min, which corresponds to a strain rate of 0.56 × 10^−3^ s^−1^.

Micromechanical properties of steel-copper wire were monitored during all periods of the study by measuring the microhardness of both the copper sheath and the steel core. To measure microhardness, an imprint was made on the sample surface under static load in accordance with GOST 9450-76. The indenter was a diamond tip in the form of a square pyramid with a square base. The load was 0.5 N for copper and 1 N for steel 1566. The hardness of wire was measured in both longitudinal and cross sections.

In subsequent processing steps and applications, steel-copper wire will undergo bending, for example, when wound on a drum during drawing, it is often studied for its behavior under such strains. Tests were carried out for three-point bending. For the bend test, 60 mm long samples were cut from steel–copper wires and reference lines that were 30 mm long were drawn on the middle part at a distance of 50 µm from each other with a microscope on the circumference. The bend test consisted of a cold strain of the pre-prepared samples on a hydraulic machine. Then, we calculated the plastic strain in the compressed and stretched areas by the change in the distance between the reference lines before and after bending.

Overall, 89 samples were strained, including at least 20 samples for each type of study.

## 3. Results

**Figure 1:**
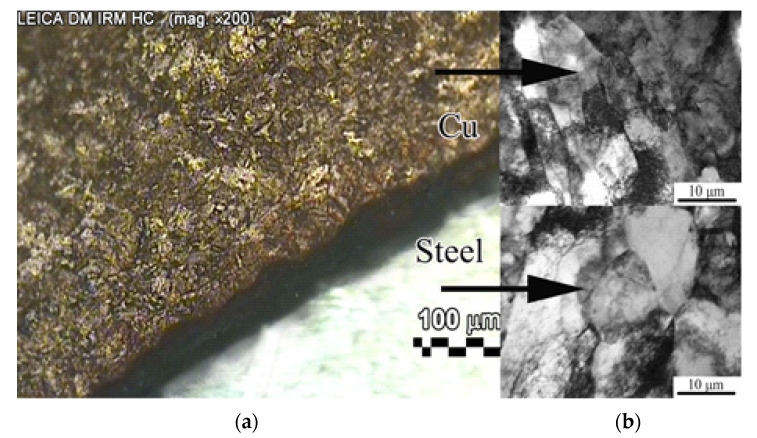
Microstructure of steel-copper wire (transversal section): (**a**) initial state; (**b**) after three passes by ECAP-drawing.

Metallographic studies of the original wire were carried out on an optical microscope so that the connection of the two metals could be observed. The study of the fine structure of the wire after strain was carried out on a transmission electron microscope (Figure 1).

**Figure 2:**
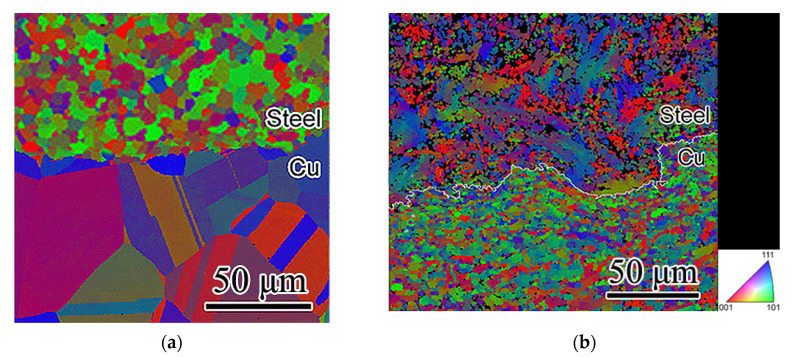
Microstructure orientation maps of steel-copper wire (transversal section): (**a**) initial state; (**b**) after three passes by ECAP-drawing.

To confirm the results obtained with a transmission electron microscope and to conduct a more detailed analysis, an EBSD analysis was used. Figure 2 shows the orientation maps of the microstructure of the steel–copper wire.

**Figure 3:**
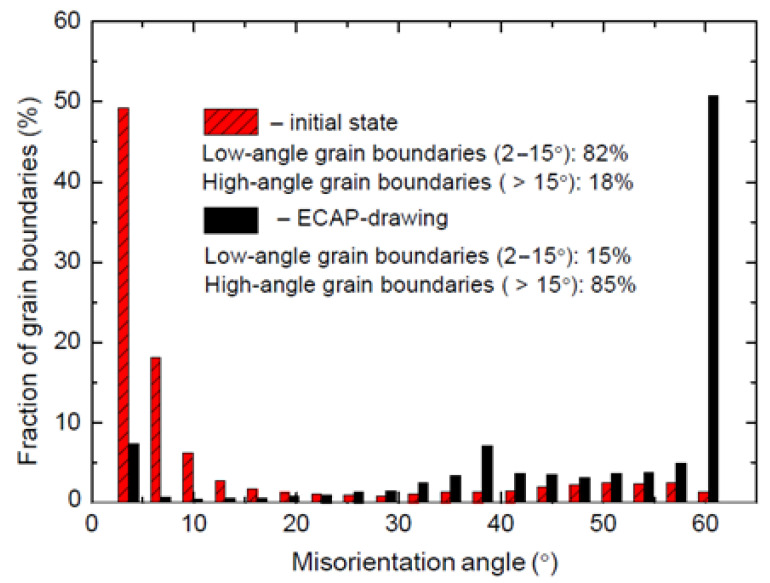
Histograms of the misorientation angles after.

Figure 3 shows the histograms of the fraction of grain boundaries plotted depending on the misorientation angle for a bimetallic wire in the initial state (highlighted in red) and after three passes of strain by the ECAP-drawing method (highlighted in black). The data in Figure 3 were obtained with the program after EBSD analysis of the cross section of the wire.

The histograms show that the proportion of high-angle boundaries in the original wire is only 18%, and after three passes of strain by the ECAP-drawing method, the proportion of high-angle boundaries increases to 85%. 

**Figure 4:**
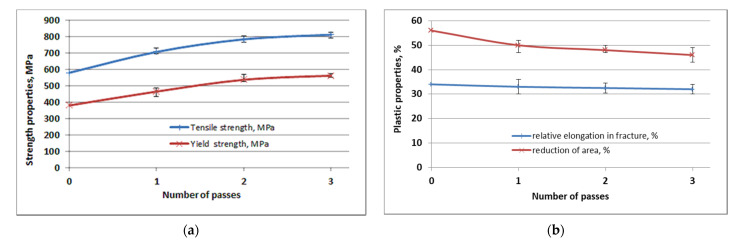
Graphs of mechanical properties with error bars: (**a**) strength properties; (**b**) plastic properties.

The tensile test determined the standard characteristics of strength and plastic properties: tensile strength (σ_B_), yield strength (σ_0,2_), relative elongation in fracture (δ), and reduction in area (ψ). Graphs of the dependence of mechanical characteristics on strain cycles are plotted, which are presented in Figure 4. In these graphs, mechanical properties are presented in the form of four values. The first value corresponds to the parameter of the bimetallic wire in its initial state. The other three values correspond to the parameters after each strain cycle.

## 4. Discussion

Microstructural studies have shown that, in the initial state, the copper shell has a coarse-grained structure with a large number of twins and an average grain size of 50 μm (Figure 1a). The initial microstructure of the steel core corresponds to the characteristic structure of high-carbon hypoeutectoid steel 1566 and is a mixture of ferrite with a large amount of perlite; the average ferrite grain size is 18 μm. After three passes, an intensive dispersion of the copper shell structure was observed; the size of the copper grains is crushed to 13 μm (Figure 1b, upper part). The fragmentation process is cumulative, i.e., the degree of grain dispersion and the thickness of the crushed layer continuously increased by passes. The core structure of 1566 steel consists of fragmented perlite sections and bent and wavy ferrite grains. The ferrite boundaries are nonequilibrium and have an entangled dislocation character. Thus, after three passes, a fragmented structure with equiaxed ferrite grains of 9 μm is formed in the steel (Figure 1b lower part). While the entire copper layer was deformed and refined even after one deformation cycle, the steel core is refined deeper with each deformation cycle. Moreover, as the number of strain passes increased, the microstructural anisotropy observed after one pass was minimized. Similar results were obtained in [57,58]. 

The initial copper shell is characterized by a chaotic distribution of grain orientations, the average grain size is ~50 μm, with a fraction of twins ~42%, the grain size of the steel core is ~18 μm, and twins are not observed, as shown in Figure 2a. After one cycle of strain, the proportion of twins in the copper increased to 48%; after two cycles, the number of twins began to decrease to 39%, and after three cycles, it decreased to 34% (Figure 2b). No twins were observed in the steel core. After three cycles of strain, the microstructure of the steel was characterized by a relatively strong contribution (111) and (001). At the interface from the copper side, the main contribution corresponds to (101) and (111). Copper showed thin bands of strain within grains as well as a large number of twins of strain origin.

After analyzing the graphs of the dependence of mechanical characteristics on the strain cycles, we can say that the strength characteristics of the steel-copper wire, presented on the graph by the curves of the tensile strength and yield strength in tension noticeably increased in comparison with the initial state. The yield strength (σ_0,2_) increases from 380 MPa in the initial state to 562 Mpa after the third pass. The tensile strength (σ_B_) after the third pass increases to 812 Mpa, and the initial state is 580 MPa. As we can observe from the curves, the main increase in strength falls on the first two passes of strain. Thus, on the first pass, the tensile strength increases by 22% and the yield strength increases by 23%.

On the contrary, plastic characteristics decrease with an increase in the number of passes, so the relative elongation in fracture decreases from 34% in the initial state to 32% after three passes. As you know, the characteristic that most fully reflects the ability of a material to strain plastic is the reduction in area in the neck area. Contraction changes from 56% to 46% in three passes. The main decrease in plasticity occurs during the first pass due to an increase in ferrite accretion, included in perlite and the crushing of cementite plates. 

Bending tests showed that, in the bimetallic wire in the original undeformed state at maximum bending, the core of steel deformed to a maximum of 20% in the tensile zone and 26% in the compression zone, and the copper sheath in the tensile zone had a plastic strain of 25%, and in the compression zone, it was 34%. Meanwhile, in the compressed area, copper and steel deformed more intensively than in the tensile zone. Bimetallic wires after three strain cycles by ECAP-drawing during the bending test showed that the copper shell in the zone of maximum tension had plastic strain of 16%, and in the compression area, it was 27%. The 1566 steel core deformed at a maximum of 18% in the tensile zone and 23% in the compressive region. An inhomogeneous stress state appeared in the steel–copper wire, which created prerequisites for changes in microhardness.

After three passes of strain by the ECAP-drawing method, a redistribution of microhardness was observed. Before strain, the microhardness values of the bimetallic wire are equal to 600 MPa for the steel core and 348 MPa for the copper shell. After strain in the copper shell, an increase in microhardness up to 663 MPa was observed near the interface of the joint with steel up to 692 MPa, which is probably related to the work-hardening of copper. The hardness in the middle and at the periphery of the shell is almost the same. The microhardness of the steel core is practically uniform over the entire section and equal to 985 MPa both in longitudinal and mean cross sections. Deviations are observed mainly near the border of the joint with steel.

It is known that it is impossible to achieve an ultrafine grain structure during conventional drawing only by increasing the total degree of strain (the number of strain passes), as this technological process is characterized by the differently aligned pattern of principal strains. In this case, tensile stresses arising in the process of strain contribute to the embrittlement of the metal during drawing. Moreover, in the traditional drawing process, the metal is riveted and acquires a fibrous structure (texture). This causes changes in physical, chemical, and especially mechanical properties of the metal, i.e., it leads to anisotropy of properties. When an equal-channel stepped die is used, all-round compressive stresses are created at all stages of strain, providing shear strain. Therefore, the mechanical properties in the longitudinal and cross-sectional sections have small anisotropy after the first strain pass. However, as strain passes increase, the anisotropy is reduced to a minimum. 

## 5. Conclusions

An important result of the study is that when combining equal-channel angular pressing with conventional drawing, the structure of bimetallic wire obtained by equal-channel angular pressing is preserved. This demonstrates the high processability and practical value of the process. The combination of ECAP with drawing makes it possible not only to harden the composition but also to simultaneously preserve important and regulated by a number of documents plastic properties of the products. This circumstance favorably distinguishes the studied method from other (traditional) types of strain treatment accompanied by modification of the structure and properties of the material. An insignificant decrease in plastic properties of steel–copper wire can be explained by the formation of a unique structure. This structure consists of ultrafine grains with large-angle non-equilibrium boundaries capable of shear. Thus, the fraction of large-angle boundaries in the initial wire is only 18%. After three passes of strain by ECAP-drawing, the fraction of large-angle boundaries increased to 85%.

The strength characteristics show good hardening of the bimetallic wire already in three passes; thus, tensile strength increases from 580 MPa in the initial state to 812 MPa after the third pass. The yield point increases from 380 MPa in the initial state to 562 MPa after the third pass. Moreover, the main increase in strength falls on the first two passes of strain. On the contrary, plastic characteristics decrease with an increase in the number of passes, but their values remain at a sufficient level for further use of the wire.

## Data Availability

Data sharing is not applicable to this article.
